# Structural characterization of Kannurin isoforms and evaluation of the role of β-hydroxy fatty acid tail length in functional specificity

**DOI:** 10.1038/s41598-020-59872-w

**Published:** 2020-02-18

**Authors:** H. Shabeer Ali, K. Ajesh, K. V. Dileep, P. Prajosh, K. Sreejith

**Affiliations:** 1 Department of Biotechnology and Microbiology, Kannur University, Kerala, 670661 India; 2 Present Address: Division of Molecular Parasitology & Immunology, CSIR-Central Drug Research Institute, Sitapur Rd, Sector 10, Jankipuram Extension, Lucknow, Uttar Pradesh 226031 India; 3 Present Address: Laboratory for Structural Bioinformatics, RIKEN Centre for Biosystems and Dynamics, RIKEN, Yokohama Campus, Yokohama, 230-0046 Japan

**Keywords:** Antimicrobials, Molecular modelling

## Abstract

The novel anti-fungal cyclic lipopeptide ‘Kannurin’ and its three structural variants produced by *Bacillus cereus* AK1 were previously reported from our laboratory. The present study reports unexplored structural variants of Kannurin those have functional benefits. Due to the difference in β-hydroxy fatty acid tail length, they are designated here as Kannurin A (m/z 994.67 ± 0.015), B (m/z 1008.68 ± 0.017), C (m/z 1022.69 ± 0.021), D (m/z 1036.70 ± 0.01), C_L_ (m/z 1040.71 ± 0.02) and D_L_ (m/z 1054.72 ± 0.01). The isoform A (m/z 994.67 ± 0.015) is the shortest cyclic form of Kannurin identified so far. In addition, C_L_ (m/z 1040.71 ± 0.02) and D_L_ (m/z 1054.72 ± 0.01) are the rare natural linear forms. The results of the antimicrobial assays deduced that the difference in lipid tail length of the isoforms contributes tremendous differences in their antimicrobial properties. The isoforms with short lipid tails (A and B) are more selective and potent towards bacteria, whereas the isoforms with long lipid tails (C and D) are more potent against fungi. The molecular dynamics studies and electron microscopic observations supported with circular dichroic spectroscopy analysis showed the structural confirmation and formation of aggregates of Kannurin in solution. The molecular dynamics simulation studies revealed that a single molecule of Kannurin makes enormous intra-molecular interactions and structural re-arrangements to attain stable lowest energy state in solution. When they reach a particular concentration (CMC) especially in aqueous environment, tends to form structural aggregates called ‘micelles’. With the structural information and activity relationship described in this study, it is trying to point out the sensitive structural entities that can be modified to improve the efficacy and target specificities of lipopeptide class of antibiotics.

## Introduction

In the modern era, lipopeptides are considered as ‘the new class of therapeutic agents’ that can be used for different clinical practices^1^. Increased demand for these therapeutic agents deserves detailed research even on their finest particulars. Kannurin, a cyclic lipopeptide belongs to the Surfactin class with slightly different heptapeptide sequence (-LLLVDLL-) was isolated from *Bacillus cereus* AK1 in our laboratory. Its broad-spectrum activity against clinically relevant yeasts and molds along with its stability over a wide range of pH and temperature make it an attractive drug candidate. Low hemolytic nature and lack of binding with melanin particles signifies it as a safe medication^2^. However, detailed structural and functional properties including anti-bacterial activity have to be explored. The present study aims to fill up the gap areas in the progression of Kannurin as a successful therapeutic agent.

While taking the entire class of lipopeptides into account, they exhibit tremendous structural diversity and mechanism of action^3^. The structural diversity of lipopeptides is mainly contributed by variations in the number, length, types and arrangement of amino acids and fatty acid tails they possess. Such structural features of lipopeptides plays an important role in determining their bioactivities^4,5^. Some of the previous reports suggested the inter connection between varying lipid tail length and change in efficacy of antimicrobial lipopeptides^6,7^. The linear forms or open conformations of lipopeptides are another set of attractive structural features. In this context, the competitive benefit of cyclic and linear forms of lipopeptides is a topic of debate^8^. In a previously reported research article, the linear forms of Arthrofactin and Surfactin produced by saponification reaction exhibited tremendous differences in their biological activities^5^. However, detailed studies on linear lipopeptides are limited due to their rare occurrence and majority of the lipopeptides reported to date are cyclic in nature.

The present study is designed to explore the structural variants of Kannurin, degree of their structural variations and the impact of those variations in their biological activities and so on.

## Results

### Isolation, purification and identification of Kannurin

Kannurin and its isoforms were purified as stated in the ‘materials and methods’. The UV spectrophotometry analysis deduced that the fractions 4, 5 and 6 have maximum absorbance values at 220 nm. Eventually, peptides were detected with the development of purple spot on sample spotted TLC plates followed by ninhydrin treatment (Fig. S1). Subsequent LCMS analysis showed that the fractions 4 and 6 contain some additional masses with m/z not related to Kannurin. Whereas, fraction 5 which contains m/z related to Kannurin was selected for further analysis.

### Identification of unexplored structural variants of Kannurin

**Figure 1:**
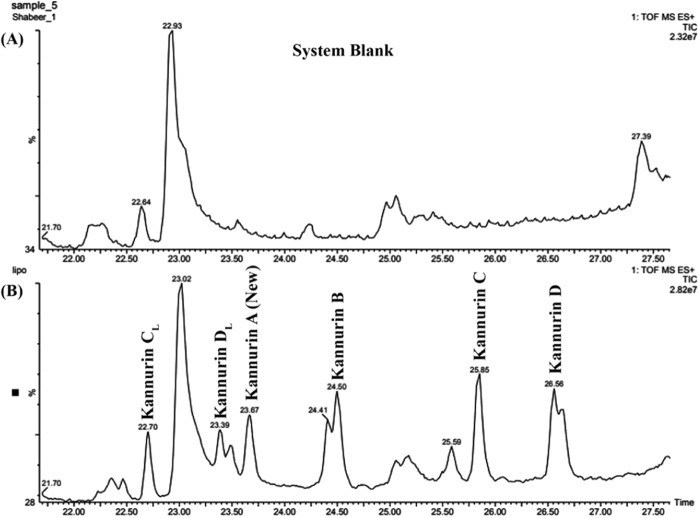
(**A**) Total ion chromatogram (TIC) of ammonium bicarbonate buffer serves as system blank that shows solvent and diluent impurities that can be omitted from the chromatogram of the purified fraction. (**B**) Total ion chromatogram (TIC) of the purified active fraction. The peaks labeled A (New), B, C and D represents cyclic Kannurin isoforms and the peaks labeled C_L_ and D_L_ represents the natural linear forms of Kannurin.

The UPLC-MS analysis of fraction 5 reproduced a characteristic elution pattern as observed earlier with approximately one minute elution time gap between the adjacent peaks. Total of 6 prominent peaks were observed in the total ion chromatogram (TIC) during 22.70 ± 0.15, 23.39 ± 0.17, 23.67 ± 0.23, 24.50 ± 0.27, 25.85 ± 0.16 and 26.56 ± 0.11 minutes of elution (Fig. 1). In the MS analysis, the corresponding TIC peaks constitutes protonated masses of m/z 1040.71 ± 0.02 (Rt = 22.70 ± 0.15 min), 1054.72 ± 0.01 (Rt = 23.39 ± 0.17 min), 994.67 ± 0.015 (Rt = 23.67 ± 0.23 min), 1008.68 ± 0.017 (Rt = 24.50 ± 0.27 min), 1022.69 ± 0.021 (Rt = 25.85 ± 0.16 min) and 1036.70 ± 0.01 (Rt = 26.56 ± 0.11 min) (Fig. S2, Table 1). Among these, the protonated masses m/z 1008.68 ± 0.017, 1022.69 ± 0.021 and 1036.70 ± 0.01 were the previously reported Kannurin isoforms^2^. At the same time, three additional prominent peaks at 22.70 ± 0.15 min (m/z 1040.71 ± 0.02), 23.39 ± 0.17 min (m/z 1054.72 ± 0.01) and 23.67 ± 0.23 min (m/z 994.67 ± 0.015) were subjected to detailed structural interpretation.

**Table 1:** Retention time of peaks observed in the total ion chromatogram and corresponding m/z.

Retention time (min)	m/z
22.70 ± 0.15	1040.71 ± 0.02
23.39 ± 0.17	1054.72 ± 0.01
23.67 ± 0.23	994.67 ± 0.015
24.50 ± 0.27	1008.68 ± 0.017
25.85 ± 0.16	1022.69 ± 0.021
26.56 ± 0.11	1036.70 ± 0.01

The retention time and m/z are recorded as the mean ± standard deviation of the values observed in 3 independent sample injections.

**Figure 2:**
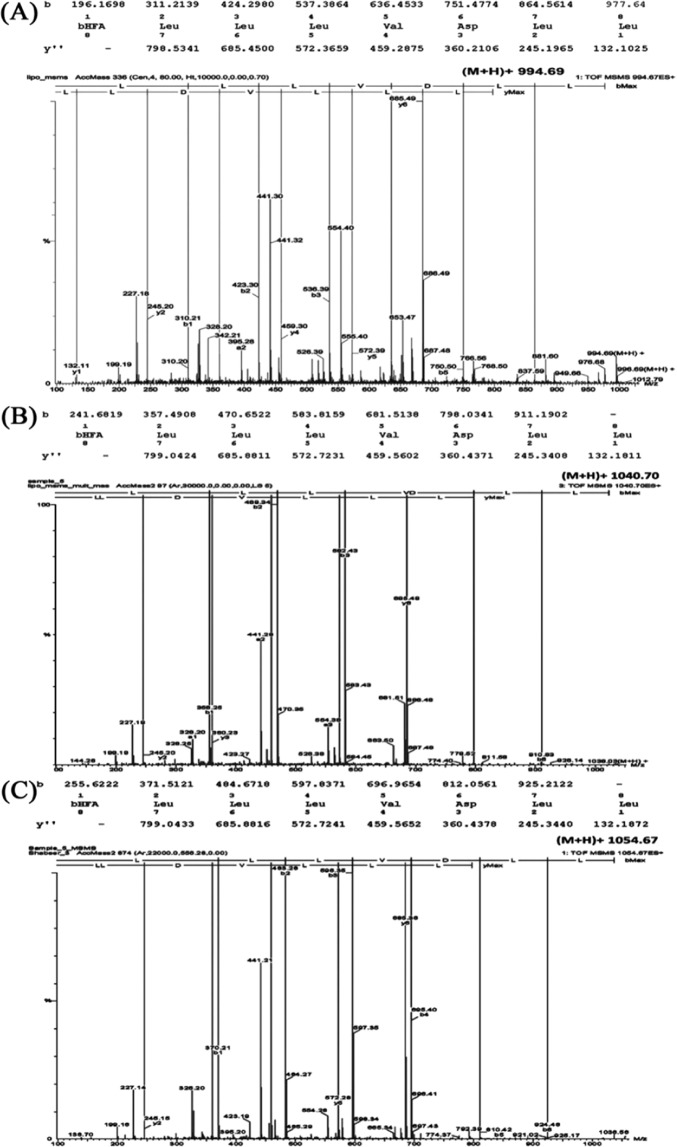
MSMS fragmentation spectrum and *de novo* sequencing of the newly found cyclic Kannurin A, m/z 994.67 ± 0.015 (**A**) and Linear Kannurin C_L,_ m/z 1040.71 ± 0.02 (**B**) and D_L_, m/z 1054.72 ± 0.01 (**C**) predicted using the peptide sequencing module (Biolynx, Masslynx 4.0.1, Waters US).

Among the protonated masses enlisted in Table 1, m/z 994.67 ± 0.015, m/z 1040.71 ± 0.02 and m/z 1054.72 ± 0.01 were not reported in the previous study. Uniform elution time interval (Table 1 and Fig. 1) and the sequential mass differences suggest their homologous nature. The m/z 994.67 ± 0.015 has a 14 Da mass difference from the previously reported Kannurin isoform (m/z 1008.68 ± 0.017). On the other hand, m/z 1040.71 ± 0.02 and 1054.72 ± 0.01 exhibits 18 Da mass differences from the previously reported Kannurin isoforms with m/z 1022.69 ± 0.021 and m/z 1036.70 ± 0.01 respectively. Interpretation of MSMS spectrum deduced that the newly found protonated masses (Fig. 2) contain a heptapeptide region with the amino acid sequence –LLLVDLL–. Due to their sequence similarity with previously reported Kannurin, the newly found isoforms were designated as Kannurin A (m/z 994.67 ± 0.015), C_L_ (m/z 1040.71 ± 0.02) and D_L_ (m/z 1054.72 ± 0.01). The alphabetical designation was based on the increasing order of the observed protonated masses. The ‘L’ in the subscript of C and D stands to denote their linear form (discussed later). The previously reported isoforms are consecutively designated here as Kannurin B, C and D (Table 2).

**Table 2:** Describes the alphabetical designations, Chromatographic and Mass spectrometry information of the different isoforms of Kannurin.

Isoform Designation	TIC Retention Time (Minutes) (Mean ± SD)	Protonated masses (m/z) (Mean ± SD)	Type	Fatty acid Tail length	Mass difference from Shortest isoform (Da)
Kannurin A	23.67 ± 0.15	994.6701 ± 0.015	Cyclic	C_12_	0
Kannurin B	24.50 ± 0.17	1008.6832 ± 0.017	Cyclic	C_13_	14
Kannurin C	25.85 ± 0.23	1022.6963 ± 0.021	Cyclic	C_14_	28
Kannurin D	26.56 ± 0.27	1036.7073 ± 0.01	Cyclic	C_15_	42
Kannurin C_L_	22.70 ± 0.16	1040.7147 ± 0.02	Linear	C_14_	18 (From cyclic C)
Kannurin D_L_	23.39 ± 0.11	1054.7286 ± 0.01	Linear	C_15_	18 (From cyclic D)

Even though the amino acid sequences are identical, a 14 Da mass difference can be observed between isoform A and B. Same mass difference can also be observed between the first four isoforms (A to D). Such mass differences could have emerged due to the difference of –CH_2_– group possibly in their β-hydroxy fatty acid tail^9^. Since the lipid tail formulae of the isoforms B, C and D had already been elucidated in our previous publication, the following lipid tail formula (C_12_H_22_O_4_) with one –CH2– group lesser than the previous isoform B was given as the post translational modification of the N-terminal leucine during the comparison of fragmentation pattern of isoform A. After this lipid tail formula modification, the fragments were seen exactly matching (Fig. 2A). In addition, appearance of a daughter ion with m/z 1012.79 (994.67 + 18 da) adjacent to the parent m/z (m/z 994.67) during MSMS fragmentation confirms the cyclic nature of the molecule. Hence it is concluded that the newly found protonated mass m/z 994.67 ± 0.015 is the shortest among the cyclic isoforms of Kannurin and designated as isoform A. It comprises 12-Carbons long β-hydroxy fatty acid tail with heptapeptide sequence exactly similar to that of the previously reported cyclic isoforms.

**Figure 3:**
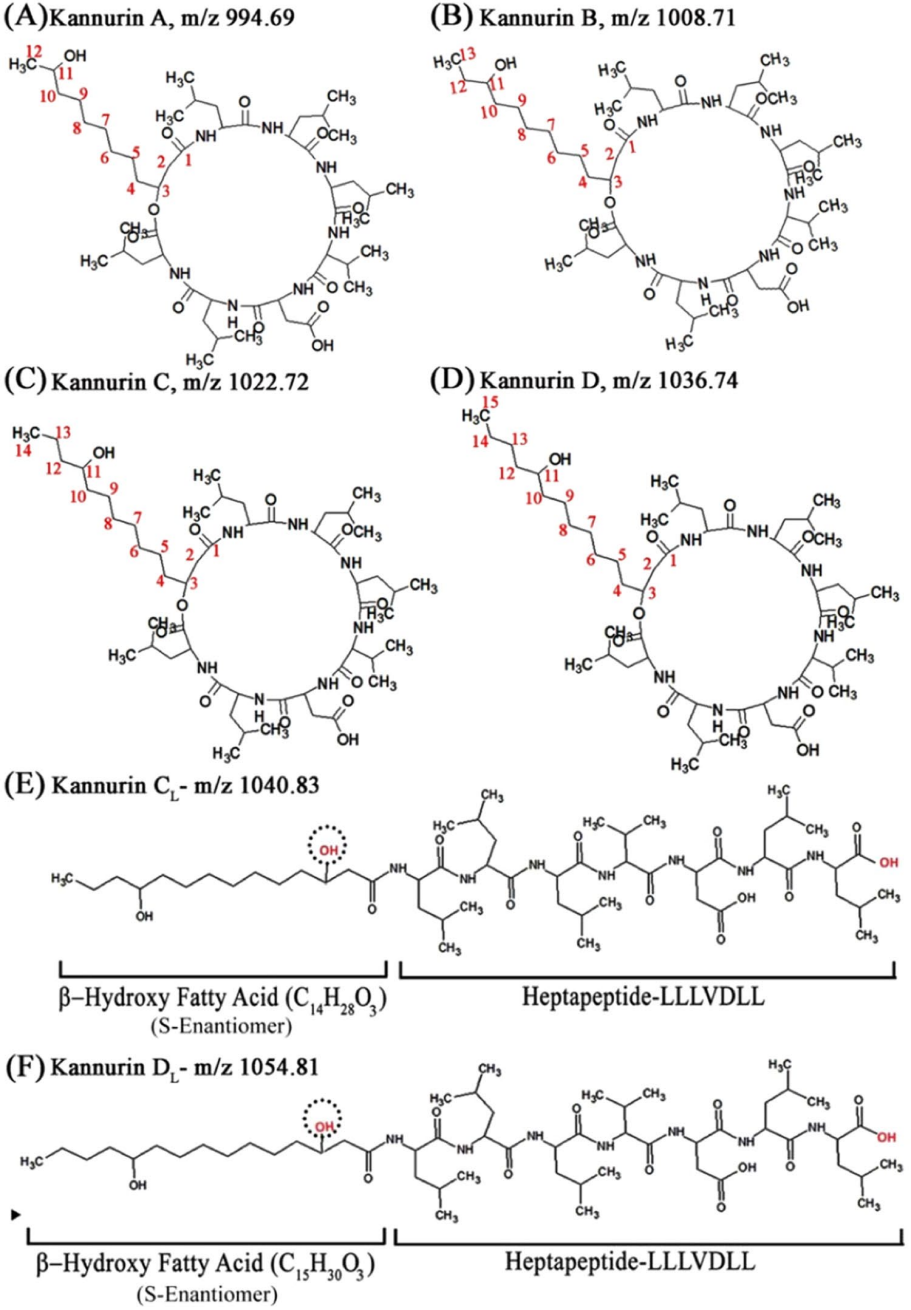
Schematic diagram of the newly found cyclic isoform A (**A**) displayed along with the previously identified Kannurin isoforms B (**B**), C (**C**) and D (**D**). The diagrams of the newly discovered linear Kannurin isoforms C_L_ (**E**) and D_L_ (**F**) are also displayed.

On the other hand, the isoforms designated C_L_ (m/z 1040.71 ± 0.02) and D_L_ (m/z 1054.72 ± 0.01) shows 18 Da mass difference from the previously reported cyclic Kannurin isoforms C (m/z 1022.69 ± 0.021) and D (m/z 1036.70 ± 0.01) respectively. Similar mass difference reported earlier in the case of linear ‘Kahalalide G’ from its cyclic counterpart ‘Kahalalide F’^10^ strengthens the assumption that the observed masses could be the natural linear forms of Kannurin. Hence the interpretation of the MSMS spectrum of C_L_ and D_L_ isoforms were performed by slightly modifying the lipid tail formulae of Kannurin C and D so as to accommodate the 18 Da excess mass differences. The lipid tail formulae that have given to interpret the MSMS spectrum of C_L_ and D_L_ are as follows, C_14_H_27_O_4_ (for C_L_) and C_15_H_29_O_4_ (for D_L_) where the β-hydroxy fatty acid possesses a free β-hydroxyl group that is not involved in esterification with C-terminal leucine. Similarly, a free carboxyl group was given to the C-terminal leucine during sequence prediction (Fig. 3E,F). Such a modified structural formula will contribute an excess 18 Da. After those modifications, the observed fragmentation patterns were seen as exactly matching with the deduced linear conformers (Fig. 2B,C). This bring into the conclusion that the isoforms C_L_ and D_L_ are the natural linear forms of Kannurin with 14 and 15 carbon long β-hydroxy fatty acid tail.

The cyclic and linear natures of the isoforms were determined by noticing the appearance of daughter ions with excess 18 Da next to the parent ion during collision induced dissociation. The basic theory states that the cyclic peptides upon fragmentation will generate a new m/z with excess 18 Da from the parent mass^11^. A new daughter ion with m/z 1012.79 (994.67 + 18 Da) was found to be generated during the fragmentation of the cyclic isoform A (m/z 994.67 ± 0.015). It may indicate that the collision induced dissociation has opened up the cyclic structure of Kannurin A (Fig. 2A). On the other hand, the MSMS spectrum of C_L_ (Fig. 2B) and D_L_ (Fig. 2C) did not showed any additional masses which indicate that the structures were already in an open conformation. With the information obtained from fragmentation prediction, the schematic sketches of the new cyclic and linear isoforms were prepared using ACD-Chemsketch software and presented in Fig. 3E,F.

### Structural studies

Molecular dynamics simulation (MDS) studies were carried out to simulate the stable conformers of cyclic and linear isoforms of Kannurin. As described in the methods section, the cyclic isoform (isoform D as the representative of the cyclic form) was constructed by adjusting the φ (phi)/ψ (psi) angles. The extensive energy minimization has produced significant changes on the initial conformation. Eventually, this energy minimized model was subjected to 30 ns MDS. The Ramachandran plot of the energy minimized model showed that all the residues are distributed in the most favored (98%) and additionally allowed regions (2%). The energy of the initial model after extensive energy minimization was dropped to −14130 kcal/mol and a series of hydrophobic interactions were also observed between L2, L3, V4 and L7 (≤5 Å). Beyond the hydrophobic interactions, two intra molecular main chain hydrogen bonds between L1-L7 and D5-L6 with in a distance ≤3.00 Å were also serves to stabilize the conformation.

**Figure 4:**
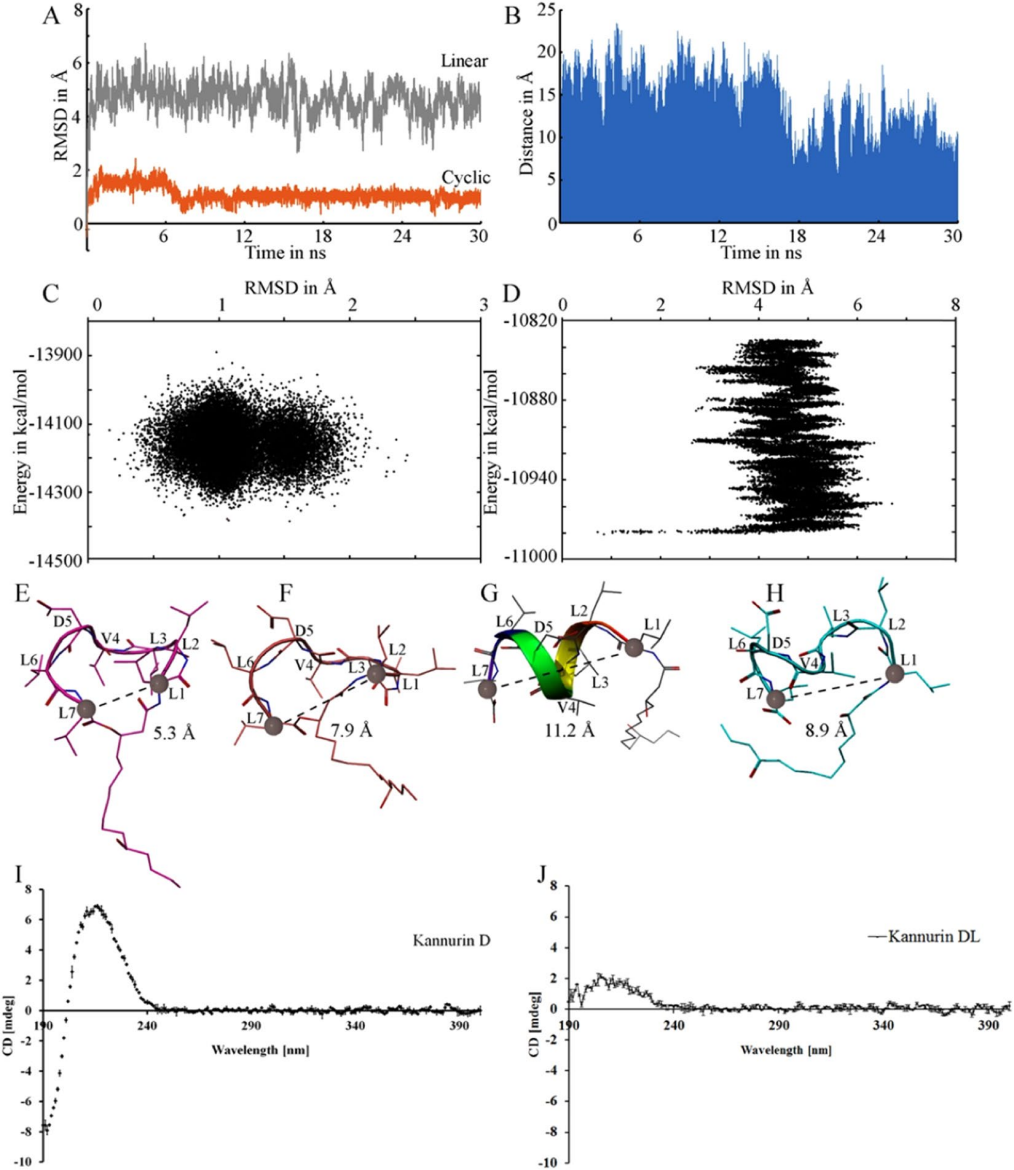
RMSD plot of cyclic (orange) and linear (grey) isoforms during 30 ns MDS (**A**). Change in distance between N and C terminal of linear isoform ‘D_L_’ during simulation (**B**). The energy vs RMSD scatter plot of cyclic (**C**) and linear isoform (**D**). The energy minimized structures of cyclic (**E**) and linear (**G**) isoforms. The lowest energy structures of the cyclic (**F**) and linear (**H**) isoforms deduced from 30 ns MDS was also shown. The results of the simulation studies are supported by the findings of CD spectroscopy analysis of Kannurin D (**I**) and D_L_ (**J**) plotted by taking the mean of three independent analysis and the standard deviation between the replicates were denoted as error bars.

The poses collected at the initial stages of MDS showed higher RMSD values when compare to those collected at later stages. The deviations were in the range of 0 to 2.5 Å as shown in the Fig. 4A. The snapshots at these regions were randomly collected and analyzed. The comparative analysis made with the initial structure and the energy minimized model deduced that the exact 2D-cyclic nature of the user constructed model was slightly distorted, which in turn reflected in the higher RMSD values at the initial stages. In the later stages, the RMSD was found to be stable at ~ 1.30 Å for the rest of the snap shots. The energy vs RMSD plot of the poses collected throughout the MDS revealed that a significant energy drop was occurred from −13900 kcal/mol (initial) to −14400 kacl/mol (final) (Fig. 4C). One of the lowest energy points was selected from the scatter plot and the corresponding structure at that point was compared with the initial structure (Fig. 4E,F). When compared with the initial structure, the maximum deviation observed was 1.73 Å and the corresponding energy was −14374 kcal/mol. The structural analysis revealed that the residues L1, L2 L3, V4 and L6 were involved in the hydrophobic interactions (≤5 Å). Similarly, two intra molecular main-chain hydrogen bonds were also observed between L6-V4 and L7-L1. Surprisingly, the side chain of D5 and the lipid tail were not involved in any type of interactions with the peptide chain. All the intermediate structures studied here are perfectly satisfying the Ramachandran plot.

3D structure of the linear isoform of Kannurin (isoform D_L_ as the representative) was constructed using Chou Fasman rule^12–14^, based on which the isoform D_L_ has a strong tendency to form alpha helix. After extensive energy minimization, it was found that the minimized structure completely satisfies the Ramachandran plot. As in the case of cyclic forms, four main chain hydrogen bonds were observed between the residues. The energy of the minimized model was −10879 kCal/mol and it was lower than that of the cyclic form.

The MDS of Kannurin D_L_ exhibited a drastic structural change (calculated in terms of RMSD) when compared to the initial model. The RMSD of the intermediate structures were distributed between 4 to 6 Å (Fig. 4A). The initial confirmation of ‘Kannurin D_L_’ generated as a result of modeling was found to be distorted during the simulation, its N and C terminals were tends to get closer as the simulation progresses. The distance between the terminals at different time points of MDS were measured and plotted in Fig. 4B. Even though there was vast structural deviation, not much energy differences were observed between the initial and energy minimized models. The energy was found to be distributed between −10820 to −11000 kcal/mol (Fig. 4D). From the different snap shots, one of the lowest energy model was selected (Fig. 4G) and compared with the initial model (Fig. 4H). The distance between N and C terminals of the energy minimized model (8.9 Å) was found to be lesser than the initial model (11.2 Å). This distance was around 7.9 Å in the case of lowest energy state of cyclic form (Fig. 4F). This indicates that attaining a cyclic conformation will allow the structures to attain stable energy state. In the energy minimized models, four possible intra molecular hydrogen bonds were observed between the residues L1-L3, L2-V4, L3-L6 and L3-L7 in a distance ≤ 3.00 Å. All the main chain hydrogen bonds at a distance cutoff ≤ 3.0 Å were shown in Table 3.

**Table 3:** Shows the residues of cyclic and linear forms of Kannurin involved in main chain hydrogen bonding within a distance cutoff ≤3 Å before and after MDS.

	Residue	Donor Atom		Accepter Atom	Residue	Distance in Å
Isoform D (Initial Structure)	L7	N	….	O	L1	2.58
	L7	N	….	O	D5	2.71
Isoform D after MD simulation	V4	N	….	O	L2	2.98
	L7	N	….	O	L1	2.72
	L7	N	….	O	V4	2.95
	L7	N	….	O	D5	2.94
Isoform E_L_ (Initial Structure)	L1	O	….	N	L3	2.90
	L2	O	….	N	D5	3.06
	L3	O	….	N	L6	2.80
	L3	O	….	N	L7	3.17
Isoform E_L_ after MD simulation	L1	O	….	N	L3	2.87
	L2	O	….	N	V4	2.94
	L3	O	….	N	L6	2.89
	L7	O	….	N	L7	2.87

**Figure 5:**
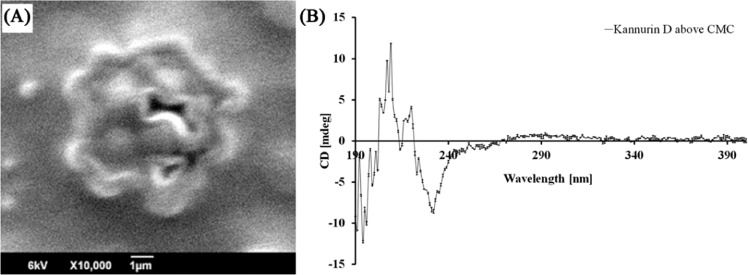
(**A**) Electron Micrograph of micelles formed by Kannurin in aqueous solution. (**B**) CD spectroscopy analysis of Kannurin to determine the formation of structural aggregates at higher concentration. The CD spectrum was plotted by taking the average of two independent replicates and the standard deviation between the replicates were incorporated as error bars.

Further, the results of electron microscopic observation and CD spectroscopic analysis also strengthen the results of MDS. The electron microscopic studies revealed that, purified cyclic Kannurin at higher concentrations tends to form aggregates in aqueous environment (Fig. 5A). The micelle formation by a group of amphiphilic molecules in solution is the general strategy to attain lowest energy state by expelling water molecules as much as possible. The hydrophobic groups of the molecule will be buried inside and the hydrophilic parts will be exposed. The bended conformation and enormous flexibility of the β-hydroxy fatty acid tail of Kannurin observed in the MDS studies indicates the possibility of a group of Kannurin molecules to form aggregates in solution by making drastic structural re-arrangements with their lipid tails. The formation of structural aggregates of Kannurin at high concentration (75 μM) in solution was observed in the electron microscopic image (Fig. 5A), which is further supported by the CD spectroscopy analysis (Fig. 5B). The formation of structural aggregates were reflected in the altered turn: random ratio (48.8: 51.2) with noisy spectrum and higher RMSD (RMSD = 84.645) (Root mean square deviation) at high concentration of Kannurin D (75 μM) (Fig. 5B). On the other hand, the CD spectrum of low concentration (10 μM) of Kannurin D (Fig. 4I) and D_L_ (Fig. 4J) exhibits a turn ratio of 100 for the former and helix: turn ratio of 6.1: 93.9 for the later. These findings supports the results of MDS which states that the peptide fragment of Kannurin D and D_L_ takes a helical conformation at their lowest energy state in solution.

### Evaluation of structure activity relationship

**Figure 6:**
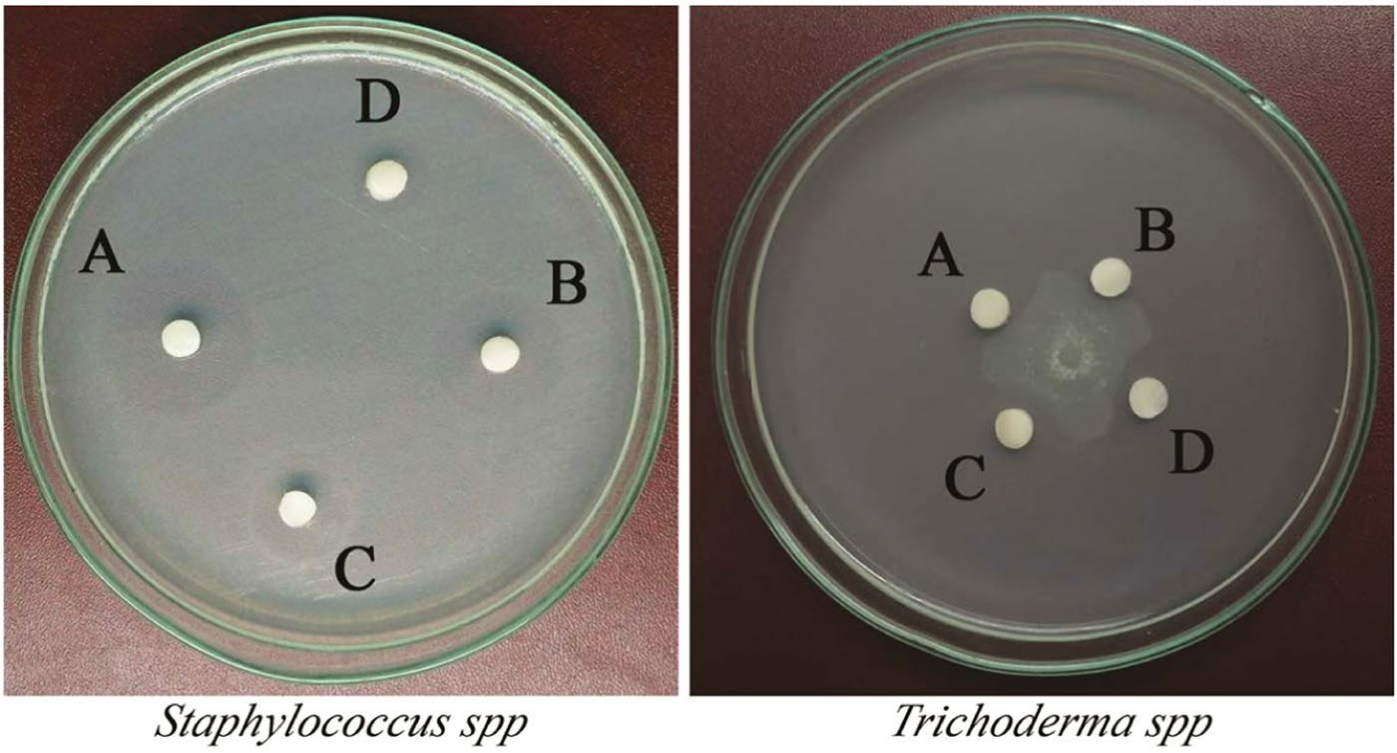
Antibacterial and antifungal activity of different isoforms of Kannurin.

The antimicrobial activity of Kannurin isoforms against bacterial (*Staphylococcus* spp) and fungal (*Trichoderma* spp) representative strains exhibited a clear structure activity relationship. Figure 6 shows the difference in inhibition zone diameter exerted by different isoforms of Kannurin against bacterial and fungal models. This observation was validated by minimum inhibitory concentration (MIC) values of the respective isoforms against the test strains. The MIC values of the isoforms A, B, C and D against *Staphylococcus* spp were 3.16 ± 0.763 μg/ml, 5.83 ± 0.28 μg/ml, 7.33 ± 0.57 μg/ml and 10.5 ± 0.5 μg/ml respectively. Whereas, the values were exactly reverse in the case of fungi, in which the MIC values of isoforms A, B, C and D were 12.5 ± 0.86 μg/ml, 9.56 ± 0.58 μg/ml, 5.73 ± 0.68 μg/ml and 4.4 ± 0.36 μg/ml respectively (Table 4). It is envisaged that the isoforms with short lipid tails (A and B) are more active against bacteria than fungi, whereas the isoforms with long lipid tails are active against fungi than bacteria. Research carried out by Malina and Shai^15^ also reported similar kind of structure activity relationship among different synthetic peptides. Since the linear forms are difficult enough to purify, their structure activity relationship is not discussed in this study.

**Table 4:** Inhibition zone diameter and MIC values of Kannurin isoforms against bacterial (Staphylococcus spp) and fungal (Trichoderma spp) test strains.

Kannurin Isoform	Organism used for the study			
	*Staphylococcus* spp		*Trichoderma* spp	
	Inhibition zone diameter (mm)	MIC (μg/ml)	Inhibition zone radius (mm)	MIC (μg/ml)
A	18.33 ± 1.52	3.16 ± 0.763	1.83 ± 0.29	12.5 ± 0.86
B	13.16 ± 1.6	5.83 ± 0.28	2.33 ± 0.57	9.56 ± 0.58
C	11 ± 1.73	7.33 ± 0.57	4.76 ± 0.93	5.73 ± 0.68
D	8.23 ± 1.53	10.5 ± 0.5	5.5 ± 0.86	4.4 ± 0.36

The values are recorded as mean ± SD.

## Discussion

In the context of antibiotics crisis, short antimicrobial peptides or lipopeptides are one of the promising candidates which can fill up the gap area to some extent. In order to better understand their molecular level of action, it is necessary to explore their structural details. Therefore, a holistic approach to study the finest structural entities of a novel class of lipopeptide antibiotic like ‘Kannurin’ is furthermore imperative in the quest of new effective agents.

This study put forward three unexplored structural variants of ‘Kannurin’. Among these, the new isoform with m/z 994.67 ± 0.015 was identified as the shortest cyclic isoform of Kannurin with 12-carbon long β-hydroxy fatty acid tail (Fig. 3A). The remaining two are the rare natural linear forms of Kannurin with protonated masses m/z 1040.71 ± 0.02 and m/z 1054.72 ± 0.01 respectively (Fig. 3E,F). With these identifications, they were alphabetically designated as Kannurin A (m/z 994.67 ± 0.015), B (m/z 1008.68 ± 0.017), C (m/z 1022.69 ± 0.021) and D (m/z 1036.70 ± 0.01). The linear forms were designated as C_L_ (m/z 1040.71 ± 0.02) and D_L_ (m/z 1054.72 ± 0.01) with a subscript ‘L’ notation to denote their linear nature.

While discussing about the application of this study, the short fatty acid-peptide conjugates possess added advantages over the long fatty acid-peptide conjugates. It was observed that the isoforms with short lipid tails are highly antibacterial, whereas the isoforms with long lipid tails are highly antifungal. The result presented in Fig. 6 indicates that the shorter isoform A could serve as a potent anti-bacterial agent and the larger isoform D as the potent anti-fungal agent. Similar result has already been reported by Malina and Shai^15^ with a combination of lipid-peptide conjugates. Hence it can also be concluded that minor variations in the lipid tail of lipid-peptide conjugates can alter target cell specificity and efficacy. Whereas the functional benefits of the linear forms of Kannurin remains to be explored. However, some linear peptides received considerable attention for their non-hemolytic nature and protective effect on RBCs against triton X-100 mediated hemolysis^16^. Due to the added advantage of such linearized forms, different organic synthesis and physical or chemical treatments has already been adopted to generate linear forms of cyclic lipopeptides^17^.

Here we theoretically describe the occurrence of natural linear forms (C_L_ and D_L_) of Kannurin which needs an insight into the bacterial non-ribosomal peptide synthesis pathway. As we understand, the cyclization of the Surfactin class of lipopeptides during their biosynthesis is catalyzed by TE domain (Thioesterase) fused to the C-terminal module of the non-ribosomal peptide synthetases (NRPSs)^18^. Within the TE domain, the cyclization of lipopeptides happens by nucleophilic attack only when an R-enantiomeric β-hydroxy fatty acid was incorporated as the tail of the peptide. If the incorporated β-hydroxy fatty acid is an S-enantiomer, then enzymatic hydrolysis will occur which in turn results in the release of a linear lipopeptide^19^. It states that the linear form of Kannurin is possibly due to the incorporation of S-enantiomeric β-hydroxy fatty acid with the heptapeptide during its non-ribosomal peptide synthesis. However, this assumption is purely based on the published literatures and additional experimental evidences are necessary to prove this hypothesis.

As in the case of other amphiphilic molecules, tremendous conformational features and structural aggregations were also observed among Kannurin molecules. MDS studies states that both the cyclic and linear forms of Kannurin can attain a stable lowest energy state even in aqueous environment. The cyclic and linear forms of Kannurin attain the stable lowest energy states by protecting their hydrophobic side chains from interaction with water molecules and also by making a series of intra-molecular hydrophobic interactions between the residues L2, L3, V4 and L7 (≤5 Å) in both linear and cyclic isoforms (Table 2). With the help of these interactions the total surface area of contact with water molecules will be significantly reduced. Similarly, the intra-molecular hydrogen bonding may also play a crucial role to stabilize the molecule. The CD spectroscopic analysis also suggests that the peptide fragment of Kannurin takes a helical conformation at lowest energy state. This is the case when Kannurin present just below the critical micellar concentrations (CMC) in an aqueous environment. As in the case of other lipopeptides, at CMC, Kannurin also form micelles (Fig. 5A). Under such conditions the intra-molecular interactions may not be the same as mentioned here, and the interactions will shift towards the formation of micelles and inter-molecular interactions will predominate. The CD spectrum of Kannurin at high concentration evidenced the formation of structural aggregates (Fig. 5B). The micelles in solution possess added biological significance over the monomer alone. The micelles believed to contribute selectivity to the antimicrobial lipopeptides towards bacterial membrane^20^.

## Conclusions

The new isoforms of Kannurin reported in this study exhibits considerable difference only in their β-hydroxy fatty acid tail. Further functional evaluation reveals a clear involvement of the fatty acid tail length in contributing improved efficacy and selectivity to the lipopeptide. Hopefully, this finding will be very useful in the future aspects of development and modification of peptide-non peptide conjugate antibiotics. This study tells to focus also on the lipid tails of other antimicrobial lipopeptides discovered so far to improve their efficacy and selectivity towards the target etiological agents. Since the lipid tails of lipopeptides play equal role in determining target specificity and efficacy, it is suggested that lipid tail modification seeks equal attention as that of peptide/amino acid modification. However, this study lacks detailed functional characterization of linear type of Kannurin, research has also been initiated in our lab in this aspect to elucidate the functional benefits of linear lipopeptides.

## Materials and Methods

### Isolation and purification of kannurin

*B.cereus* AK1 was inoculated in to the fermentation broth (3% peptone, 0.5% NaCl and 0.5% yeast extract, pH = 7)^21^ and incubated at 37 °C for 72 h with continuous agitation (150 rpm). After incubation, the cultures were centrifuged at 15000 g for 20 minutes and filtered through membrane filter (0.2 μm). The cell free supernatant was precipitated by adding 1% CaCl_2_ (w/v). The precipitates were collected by centrifugation, re-dissolved in Tris-EDTA buffer (pH = 8.0) and dialyzed overnight against sodium phosphate buffer (pH-7.0) using a benzoylated dialysis tubing (Pore size 2000 NMWCO). 75% (v/v) ethanol was added to the dialysate, kept at 4 °C for 6 hours and the precipitates were removed by centrifugation. The supernatant was dried under vacuum, re-suspended in minimal volume of water and precipitated using Hydrochloric acid (pH 3). The precipitates were collected by centrifugation, re-dissolved in 0.05 M ammonium bicarbonate buffer and dialyzed overnight against de-ionized water. The dialysate was then subjected to gel filtration/size exclusion chromatography.

### Size exclusion chromatography

Sephadex G-25 column matrix was prepared as per the manufacturer’s instruction (Sigma Aldrich, USA) and packed in a glass column (120 × 1.5 cm, Bio-Rad). The loaded sample volume was 1/5^th^ of the total column matrix volume. The elution was performed using 0.05 M Ammonium bicarbonate buffer with a flow rate of 30 ml/min. Fractions of 2 ml per tube was collected. Based on the antibacterial activity observed, the active fractions were subjected to UPLC-MS/MS analysis.

### LCMS/MS analysis

The active fractions were subjected to ‘Ultra performance liquid chromatography’ (UPLC) (Acquity-UPLC, Waters). C18-Reverse phase column with particle size of 1.7 μ and column dimension 2.1 × 50 mm was used for the study. The sample was eluted using mobile phase A (0.1% Formic acid in water) and B (100% Acetonitrile) with a gradient elution starting from 100% A-0% B (initial) to 0% A-100% B (Final) with a flow rate of 0.6 ml/min over a 20 minutes run. The column and sample temperatures were kept at 25 °C throughout the analysis. The eluted fractions were then subjected to mass spectrometry (MS) analysis using Xevo-G2S-QTof MS/MS system coupled with the Acquity UPLC unit. The MS analysis was performed in positive ESI mode with a capillary voltage of 3000 V and sampling cone voltage 30 V. The source and desolvation temperatures were kept at 150 °C and 200 °C respectively. The cone and desolvation gas flow was set to 80 and 1000 L/Hr respectively. The desired masses were then subjected to collision induced dissociation in the same instrument. The collision energy was standardized by applying a range of energy from 10 V to 70 V. The sequence was compared using the peptide sequencing module (Biolynx, Masslynx 4.0.1) provided by Waters (US).

In order to cross verify the occurrence of linear forms of Kannurin, the whole experiment was conducted in the absence of saponifying agents and hydrolytic acids. The MS instrument parameters were also strictly controlled to prevent in-source fragmentation and false interpretation. The capillary voltage was brought down to 1500 V from 3000 V. Similarly, the sampling cone voltage was reduced from 30 V to 15 V. The parameters were adjusted to a threshold by ensuring that it will not affect the peak shape, response, ionization and intensity of the data. The reproducibility of the LCMS data was ensured by performing the repeated analysis of the same molecules purified at different time points. Standard deviations of the retention time and protonated masses were calculated from the results obtained from multiple injections.

### Molecular modeling studies of Kannurin

Molecular modeling studies were carried out to predict the structural features and stable conformers of cyclic and linear forms of Kannurin. The cyclic isoform (isoform D as the representative) of Kannurin was constructed using Schrodinger Maestro (9.0.1)^22^ by carefully adjusting the φ (phi)/ψ (psi) angles. Later the N and C terminal of the peptide was prudently connected through an ester bond with a 15 carbons long β-hydroxy-fatty acid (Kannurin D). The linear form of Kannurin was constructed based on the secondary structure predicted through Chou Fassman rule. The user constructed cyclic and linear forms were subjected to extensive energy minimization using OPLS-2005^23^ force field. Structural distortions were prevented by applying dielectric constant cut off corresponding to water. The cut off applied for van der Waals, electrostatic and H-bond were 7.0, 12.0 and 4.0 Å respectively. The Polak-Ribier Conjugate Gradient method with maximum iterations 100,000 were given for the minimization. The energy and three dimensional structures were compared before and after the energy minimization to understand energy drop and structural changes. The minimized models were subjected to molecular dynamics simulation (MDS) to study the physical movement and structural features of the cyclic lipopeptide in solution. The MDS was performed using Desmond at 300 K with NPT ensemble^24^. The temperature and pressure of the system was controlled by Nose-Hoover chain (thermostat) and Martyna-Tobias-Klein (barostat) methods respectively. Prior to MDS, the energy minimized model was prepared by soaking it in an orthorhombic water box containing 1377 TIP3P water molecules and ions (to neutralize the system). OPLS-2005 force field was used to perform the 30 ns MDS. Snapshots were collected at every 1.2 ps, superimposed with the initial model and the root mean square deviations (RMSD) for each poses were calculated. The corresponding energy at different time point was also recorded to plot the energy versus RMSD graph.

### CD Spectroscopy analysis

The CD spectroscopy analysis of was performed using JASCO CD J-815 instrument. The spectra were acquired using a quartz cell with cell length of 10 mm and the data were collected over a wavelength range of 400–190 nm at 1 nm interval. The Kannurin isoforms were scanned at 10 and 75 μM concentrations to distinguish the conformational features at high and low concentrations. Samples purified at different time points were submitted as independent replicates. The CD spectrum presented in the results section is the mean of independent replicates and the error bars indicates standard deviation between the replicates.

### Scanning electron microscope of micelles

The solution containing critical micellar concentration (CMC) of Kannurin was coated as a thin film on the surface of a 5 mm^2^ glass slide and air dried. The specimen was then placed on the copper grid and coated with Gold for 45 seconds to get a thickness of about 40‐60 nm using JFC-1600 auto fine coater. The developed specimen was then observed using the scanning electron microscope (JEOL, JSM 6390) and the images were collected at appropriate magnification.

### Antibacterial and antifungal assay

The initial screening to distinguish the differences in antimicrobial activity was determined by Kirby-Bauer disk diffusion method. 15–20 μl of the purified, concentrated cyclic isoforms were loaded on to the sterile paper disk and placed on the surface of Muller-Hinton agar plates seeded with 0.5 McFarland solution of *Staphylococcus* spp. In order to study the antifungal activity, spore suspension of *Trichoderma* spp was prepared and point inoculated at the center of Sabouraud’s dextrose agar (SDA) plate. After 72 hours of incubation, paper discs loaded with Kannurin isoforms were placed distant to the growing fungal mat. In both the cases, the inhibition zone diameter was recorded. Further, the MICs of each isoforms were also determined by broth micro-dilution method as described earlier^25^. In order to ensure statistically significant result, both the experiments were run as independent triplicates in which the Kannurin molecules were purified at different time points.

### Statistical analysis

Purification, analysis and other functional evaluation studies (antibacterial and antifungal) were carried out as independent triplicates to ensure reproducibility and authenticity of the observed results. The statistical data processing were carried out by Microsoft excel, Graphpad Prism (Version 5) and Origin pro 8.0 software.

## Supplementary information

Supplementary information
