# Supplementary material for: Structural characterization of Kannurin isoforms and evaluation of the role of β-hydroxy fatty acid tail length in functional specificity
Source: Sci Rep. 2020 Feb 18;10:2839. doi: 10.1038/s41598-020-59872-w (PMC7029025; doi:10.1038/s41598-020-59872-w)
Supplement: Supplementary file 1 — Supplementary information [file 41598_2020_59872_MOESM1_ESM.pdf]

Structural characterization of Kannurin isoforms and evaluation of the role of β-hydroxy fatty acid tail length in functional specificity

Shabeer Ali H.[1,2], Ajesh K.[1], Dileep K.V.[1,3], Prajosh P.[1], Sreejith K.[1]*

[1]Department of Biotechnology and Microbiology, Kannur University, Kerala, India-670661.

[2]Current address: Division of Molecular Parasitology & Immunology, CSIR-Central Drug Research Institute, Sitapur Rd, Sector 10, Jankipuram Extension, Lucknow, Uttar Pradesh, India-226031.

[3]Current address: Laboratory for Structural Bioinformatics, RIKEN Centre for Biosystems and Dynamics, RIKEN, Yokohama Campus, Yokohama, Japan 230-0046.

*For correspondence - sreejithkrishnan@hotmail.com

Phone : +91 9446870675

Supplementary Figures

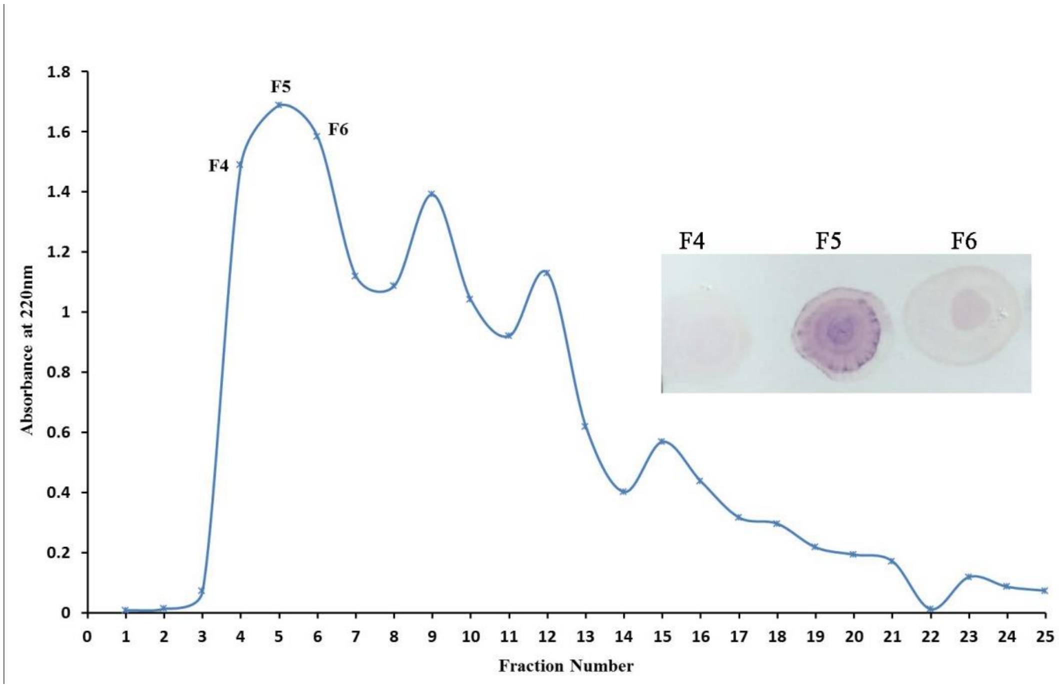

Figure S1. Shows the UV absorbance values at 220nm of the fractions obtained after gel filtration column. The inset shows the result of Ninhydrin test performed on TLC plates loaded with the Sephadex-G25 column fractions.

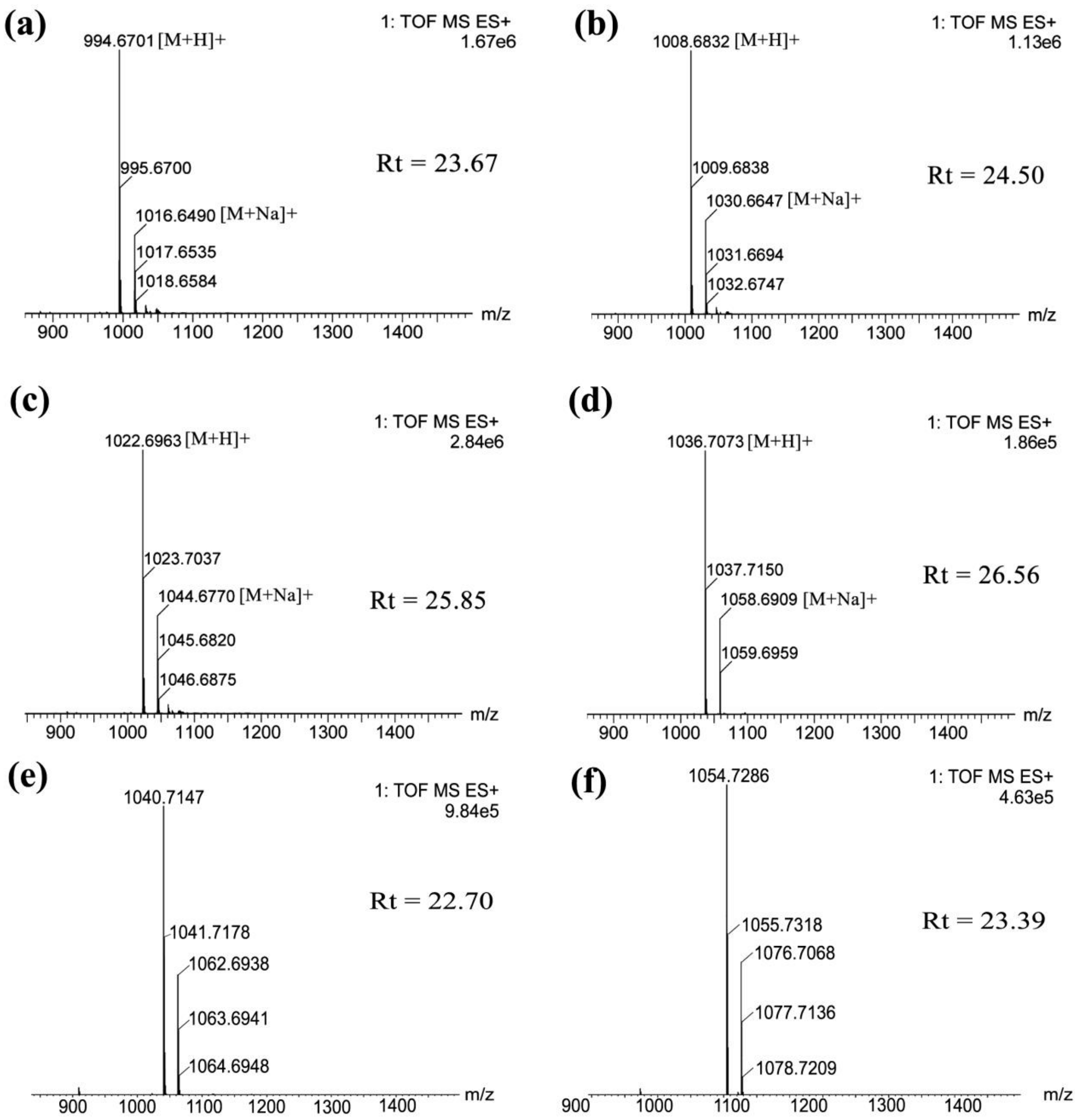

Figure S2. The protonated masses of the corresponding TIC peaks. The retention time of the peaks are given in the inset.
